# Neurosyphilis-Induced Psychosis in Europe: A Systematic Review of Case Reports

**DOI:** 10.3390/pathogens13110959

**Published:** 2024-11-04

**Authors:** Adam Jarocki, Kinga Klimczyk, Monika E. Łysakowska, Filip Bielec, Dorota Pastuszak-Lewandoska

**Affiliations:** Department of Microbiology and Medical Laboratory Immunology, Medical University of Lodz, 92-213 Lodz, Poland; adam.jarocki@student.umed.lodz.pl (A.J.); kinga.klimczyk@student.umed.lodz.pl (K.K.); filip.bielec@umed.lodz.pl (F.B.); dorota.pastuszak-lewandoska@umed.lodz.pl (D.P.-L.)

**Keywords:** syphilis, *Treponema pallidum*, psychosis, neurosyphilis, systematic review

## Abstract

The tertiary stage of syphilis appears to be the most diverse of the three recognized, with a number of cardiovascular, gummatous, neurological and psychiatric symptoms. This systematic review’s aim is to analyze cases of organic psychoses secondary to tertiary syphilis, inspect the diagnostic procedures and assess the effectiveness of treatment. Case studies from Pubmed and MEDLINE Ultimate were sought out with the Boolean expression ‘((neurosyphilis) OR (syphilis) OR (*treponema pallidum*)) AND (case report) AND ((psychosis) OR (psychotic))’ to later undergo screening for exclusion criteria (according to the Preferred Reporting Items for Systematic reviews and Meta-Analyses (PRISMA) 2020 guidelines). Each report was accepted independently by two authors. Case reports were later appraised using the JBI Critical Appraisal Checklist for Case Reports. Finally, 11 case reports were included in the study. The most frequently reported psychiatric symptoms were delusions (91%) and hallucinations (81%), disorientation (about 42%) and sleep disturbances and memory loss (36%). Several inconsistencies in diagnostic processes were found in some of the case reports, while treatment procedures were more in line with the guidelines. A disease as complex as neurosyphilis requires great awareness and cooperation between various medical specialties. Despite its mimicry and variability in presentation, the discussed case reports prove that it can be successfully dealt with.

## 1. Introduction

Syphilis is a sexually transmitted disease caused by *Treponema pallidum* subsp. *pallidum*, presenting with a wide range of signs and symptoms. For hundreds of years, this enigmatic disease maimed and killed, and the combination of the image of syphilis with national prejudices resulted in the disease being attributed to disliked neighbors, inspiring names such as Neapolitan/French/Polish disease and even Christian disease [1]. Even now, in the antibiotic era, syphilis was observed to surge in incidence by as much as 34% in the span of one year (European Union Member States), as per the European Centre for Disease Prevention and Control (ECDC)’s Syphilis Annual Epidemiological Report 2022. In the same time frame, the number of member states reporting early latent syphilis diminished in favor of primary or secondary stages; however, details on the clinical stage of syphilis were provided only by 15 countries for 27% of cases (*n* = 9 382), which may distort the overall epidemiological situation [2].

Epidemiological data from various regions underscore the global burden of syphilis. For instance, in China, studies have shown significant predictors of seronegative conversion after treatment, highlighting regional differences in disease management [3]. In response to the growing epidemic, Brazil declared a national syphilis epidemic in 2016 and launched the ‘No to Syphilis’ Project, marking a significant effort in tackling the issue. This project and other global initiatives reflect the urgency of addressing syphilis, as case numbers continue to rise worldwide [4,5]. In Europe alone, the number of reported syphilis cases has been steadily increasing since 2010, with men who have sex with men (MSM) and co-infection with HIV being particularly prevalent in the epidemiological landscape [5]. Such data emphasizes the relevance of this disease within global public health.

Neurosyphilis, the central theme of this manuscript, further complicates the picture. As syphilis progresses, it can invade the central nervous system, leading to severe and often irreversible damage. Neurosyphilis remains a challenge due to its wide range of clinical manifestations, including psychiatric symptoms and even psychosis, complicating diagnosis and treatment efforts [6]. The global public health community continues to work on interventions to address both syphilis and its neurological complications, but significant gaps remain in understanding and managing this complex condition. Recent scientific publications from 2021 to 2024 have provided new insights into the epidemiology, diagnosis, and treatment of syphilis, contributing valuable knowledge to the ongoing fight against this ancient disease [7,8].

The significance of syphilis as a global health issue cannot be overstated. Recent reports highlight the efforts of health systems across the world, including Europe, North America, and Latin America, to combat this resurgence. However, a coordinated global response is needed to control the spread of syphilis and prevent its progression to severe stages, such as neurosyphilis [9,10].

### 1.1. Clinical Manifestations

Syphilis presents with primary and secondary stages and becomes asymptomatic, being considered ‘early latent’ for the first year after inoculation and ‘late latent’ when the time from acquisition exceeds one year [2]. *T. pallidum* infection can progress into the tertiary stage within years or decades after the entry of pathogens [6].

The primary stage of syphilis is marked by the appearance of a chancre, a clean-based, painless lesion, mostly in the anogenital region usually within 3 weeks after transmission, although the incubation period for *T. pallidum* is reported to be 10 to 90 days. The lesion lasts 3–6 weeks if untreated but only a few days if early treatment is employed (preferably with benzathine penicillin). It is recommended that any ulcer in the anogenital area should be approached as if it were syphilitic, unless it can be disproven [6,11]. Dissemination of *T. pallidum* takes place in the primary stage, paving the way for the secondary stage of the infection [12].

The secondary stage is typically marked by the appearance of a macular rash, including on the soles and palms, which then spreads with 90% chance of mucous membrane involvement, though the degree of spread and the forms of the lesions are highly variable. Lesions present on mucosae may take on the appearance of *condylomata lata*, moist, wide-based and highly infectious warts [6,11,13]. Secondary lesions, if left untreated, persist for weeks to months, with 25% of patients relapsing within the first year of the early latent stage [6,14]. Bacteremia, possible hepatosplenomegaly, lymphadenopathy, hepatitis, and neurological symptoms occur. Dermatological lesions pose a great challenge for the physician seeing the plethora of possible signs, similarity to other illnesses and the fact that none of the skin lesions leave scarring after they disappear, all add to difficulty in diagnosis [6,11].

**Figure 1:**
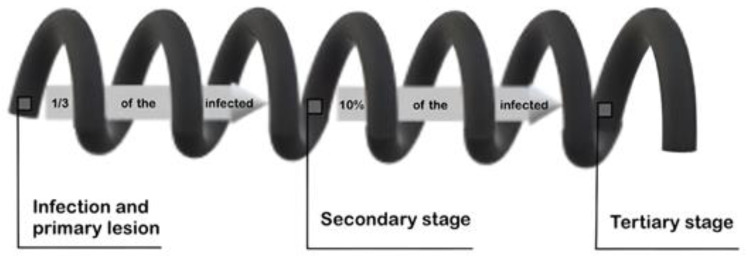
Likelihood of reaching each stage of syphilis. The arrows present the likeliness of developing the stage of the disease. Figure made by the authors.

The tertiary stage appears to be the most diverse of the three, as it can have a number of cardiovascular, gummatous or neurological (‘neurosyphilis’, ‘NS’) manifestations, including even psychiatric symptoms [2]. It is worth noting that not all infections of *T. pallidum* end in the tertiary stage. Only in one-third of infections is *T. pallidum* able to disseminate throughout the body, causing the secondary stage [11], and in around 30% of secondary infections [11] the pathogens manage to hide from the host’s immune response long enough for the disease to reach the tertiary stage (Figure 1) [11,12].

### 1.2. Virulence

*T. pallidum*’s complex pathogenicity results from numerous adaptations and key morphological and metabolic characteristics. The pathogen is often regarded as Gram-negative because of the presence of outer and cytoplasmic membranes, as well as a layer of peptidoglycan between them, although some notable differences are present in *T. pallidum*, one of which being the lack of lipopolysaccharides (LPSs) [12,15]. This antigen, a hallmark of Gram-negative bacteria, is not coded in the pathogen’s modest genome of about 1 million base-pairs; it also does not code enzymes such as catalase, superoxide dismutase or those of the Kreb’s cycle or β-oxidation [12,16] or several other outer membrane proteins (OMPs). In fact, a lack of OMPs may be considered an adaptation of the bacteria whose outer membrane is observed to have OMP density as low as 1% of that of *E. coli* cells [12,17]. The low density of proteins in the membrane translates to the bacteria’s exceptional ability to hide from the host’s immune system. In vitro studies confirm that many *T. pallidum* cells immobilized in a medium remain unresponsive to sera containing specific antibodies and require significant time or additional treatment to be killed by them [18]. *T. pallidum*’s tropism towards certain niches such as nerves, hair follicles and erector pili muscles allows further evasion of the immune response [19]. Masterful hiding comes at the expense of a slow metabolism; OMPs, which the bacterium does not have, under standard conditions serve to accumulate resources necessary for functioning, which is why *T. pallidum* multiplies in up to 35–40 h [12]. Another key component of treponemal pathogenicity is the endoflagellum, which is quite unique, as it rests in the periplasmic space between the outer and the cytoplasmic membrane, and along with the corkscrew shape of the bacterium allows for passage through viscous media and dissemination throughout the body [12,13,15].

### 1.3. Vaccine

No vaccine is yet available against *T. pallidum* likely due to its low OMP density, as OMPs are the usual targets for immunization. Only one successful vaccine was formulated and tested on rabbits in 1973. It was the only one, thus far, to prevent infection, but it required a course of 60 injections, and therefore it cannot be considered viable [20,21]. Research on *T. pallidum* immunization is ongoing and provides valuable data on the relationship between the antigens used and the results obtained. So far, various antigens have been found to reduce the dissemination of the bacteria, lower the bacterial burden in lesions or decrease lesion volume when used for immunization [8,22].

### 1.4. Psychosis

CNS infection of *T. pallidum* etiology is known to be a potential cause of ‘organic psychosis’, a psychotic syndrome that is secondary to somatic conditions and one that is not related to mental or behavioral disorders. As per the *International Classification of Diseases 11th edition* (*ICD-11*), a ‘psychotic syndrome’ means the presence of hallucinations and/or delusions. A ‘Secondary psychotic syndrome’ bears the code 6E61 [23,24]. There seems to be a significant difference in the perception of psychosis itself between the *ICD-11* and the *Diagnostic and Statistical Manual, 5th edition*; in the latter, the boundaries of psychosis are not as rigid, allowing for diagnosis even in cases without hallucinations or delusions but presenting with disorganized thought or behavior and negative symptoms [25].

This article’s aim is to analyze cases of organic psychoses secondary to tertiary syphilis, look for possible clues in diagnosis, inspect the diagnostic tools and determine the immediate effectiveness of treatment.

## 2. Methodology

This study was conducted according to the systematic review methodology based on the PRISMA 2020 guidelines [26]. The study protocol, registered in the OSF Registries database, is available at https://doi.org/10.17605/OSF.IO/TS4KG (accessed on 6 September 2024). Preferred Reporting Items for Systematic reviews and Meta-Analyses extension for Scoping Reviews (PRISMA-ScR) Checklist in Supplementary Materials (Table S1). 

### 2.1. Data Collection

Case studies from PubMed and MEDLINE Ultimate were sought out with the Boolean expression ‘((neurosyphilis) OR (syphilis) OR (*treponema pallidum*)) AND (case report) AND ((psychosis) OR (psychotic))’ to later undergo screening for exclusion criteria. It is important to point out that terms pertaining to Europe were not used in the Boolean search, as they were found to be unreliable, falsely excluding many reports of European origin.

### 2.2. Inclusion and Exclusion Criteria

For a case report to be accepted for further analysis, the following criteria had to be met:

- The case (the patient) was of European origin;
- The case presented a neurosyphilis diagnosis;
- Psychotic symptoms had to be present;
- Psychotic symptoms described had to be attributed to the neurosyphilis diagnosis.

As for exclusion criteria, they included the following:

- Psychosis not secondary to neurosyphilis, but primary to another diagnosis such as schizophrenia, stupor or bipolar disorder;
- Articles in a language other than English;
- Neurosyphilis without psychotic symptoms;
- Non-European origin of the case.

The analysis was restricted to the cases reported in Europe to limit the variation in the clinical presentation of neurosyphilis. Ease of traveling within Europe makes it likely for a clinician to encounter a patient from the continent treated with antibiotics recommended in Europe, and such treatment may impact the clinical presentation. Previous antibiotic therapy could influence the patient’s immune status and possibly change the course of infection. To avoid the influence of other variables, the authors decided to study syphilis cases that were reported in one (relatively consistent in terms of antibiotic therapy) area.

Each report was accepted independently by two authors of this review. Notes on each report were made in a spreadsheet; they included age, sex, psychiatric and neurological symptoms, diagnostic procedures, treatment and patient follow-up. Case reports were later appraised using the JBI Critical Appraisal Checklist for Case Reports.

## 3. Results and Discussion

**Figure 2:**
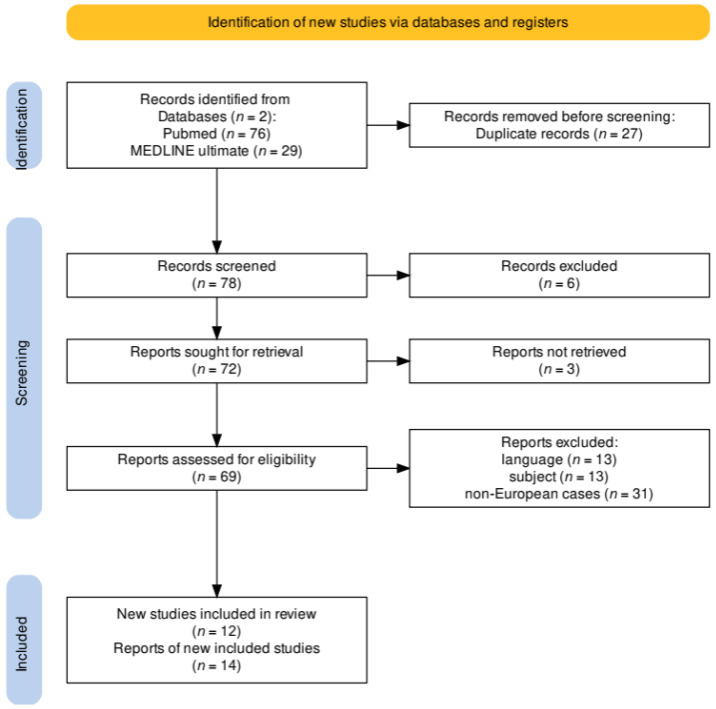
PRISMA flow diagram of this review’s systematic research.

Initially, 105 case reports were found in the databases, of which 27 duplicate records were removed, leaving the authors with 78 records to screen. Six of those were at that point excluded due to incorrect topics or formats, and another three could not be retrieved. From the remaining 69 reports, the authors excluded 13 in languages other than English, 31 cases of non-European origin of the disease and 13 that met the remaining exclusion criteria. The remaining 12 studies contained 14 case reports. The PRISMA flow diagram (Figure 2) illustrates the selection process. For the titles and authors of the accepted case reports, see Table 1.

Retrieved case reports underwent further analysis using the JBI Critical Appraisal Checklist for Case Reports, which led to the exclusion of three case reports due to insufficient data on antibiotic treatment. Criteria such as unanticipated events or long-term post-intervention conditions were not used as exclusion criteria, seeing how long it takes to control syphilis and what this article’s goals are. Finally, 11 case reports remained.

For the detailed JBI Checklist, see Table 2.

### 3.1. Study Patients

The median age of patients was 40, the oldest being 90 [36] and the youngest 30 years old [37]. The ratio of female to male patients was 3 to 8.

All patients met the criteria of the *ICD-11* 6E61 (secondary psychotic syndrome) diagnosis; there were always hallucinations and/or delusions present, the symptoms were severe, and neurosyphilis was detected and other potential causes excluded [23]. In addition to the international, formal classification, there are other, somewhat historical, terms in the medical nomenclature associated with neurosyphilis, like ‘general paresis of the insane’ (‘GPI’, ‘dementia paralytica’) and ‘tabes dorsalis’ (locomotor ataxia). GPI concerns the brain of a late-stage patient with neurosyphilis, associated with forgetfulness, confusion, deteriorated behavior, disinhibition and memory issues [39,40]. The term ‘tabes dorsalis’ describes the degeneration of neurons in the dorsal column of the spine, which causes loss of sensation, uncoordinated movements and incontinence [41].

### 3.2. Diagnosis of Neurosyphilis

The first step towards achieving the resolution of symptoms or any relief to the patient is a proper diagnostic regimen. However, although the gold standard in *T. pallidum* examination is dark-field microscopy, with a high specificity of 82–100% and a medium-to-high sensitivity of 39–81%, it cannot be applied in neurosyphilis, as there are no lesions to acquire live specimens from [14]. To make matters worse, in vitro cultivation of *T. pallidum* is difficult [42], and in vivo cultivation, using rabbits, is expensive and rather used for scientific purposes [43]. None of these methods were used in the reviewed case reports.

There are various serological tests, divided into two groups: treponemal and non-treponemal. Non-treponemal tests (NTTs) include Rapid Plasma Reagin (RPR), the Toluidine Red Unheated Serum Test (TRUST) and Venereal Disease Research Laboratory (VDRL). They take their names from the fact that the reagent used is a mixture of lecithin, cardiolipin and cholesterol, mimicking actual bacterial antigens. The most common treponemal tests (TTs) are the *Treponema pallidum* Hemagglutination Assay (TPHA), Treponema pallidum Particle agglutination Assay (TPPA), Fluorescent Treponemal Antibody Absorption Test (FTA-ABS) and ELISA; those tests operate on recombinant antigens of *T. pallidum* and are more sensitive than their non-treponemal counterparts [6,11,14,43,44].

Despite such a relative abundance of available diagnostic methods, they all have severe shortcomings, especially when testing for tertiary syphilis:

- The CSF-VDRL (cerebro-spinal fluid VDRL) test’s sensitivity is reported to range from an unsatisfactory 30% to a somewhat acceptable 70%. CSF-RPR is considered even less sensitive [11,43].
- Non-treponemal tests are likely to give false-positive results, which means that such tests alone do not provide concrete proof of infection [11,43,45].
- Serum TTs remain positive for the rest of the patient’s life, rendering them useless for monitoring [11,14,46].
- About 15–20% of patients may remain serofast, meaning that their NTT titer will reach a low plateau and stop decreasing, even after re-treatment. Such serological non-responsiveness has been linked to weak reactivity of the patient’s immune system to the TpN47 antigen of *T. pallidum* pre-treatment [47]. Serofast patients should be periodically followed up [6,14].
- Finally, no serological test is able to distinguish *T. pallidum* subspecies (*pallidum*, *pertenue* and *endemicum*) due to their genomic identity reaching 99.7–99.8% [10,11,12,14,48,49].

The basis for the initial diagnosis of neurosyphilis is serological tests, along with the patient’s clinical presentation and history, especially regarding sexual relations. There are three main approaches to testing for syphilis, all of which begin with serum testing:

- The traditional algorithm uses an NTT for screening and a TT for confirmation. This method is more cost-effective but less sensitive [11,43,44].
- The reverse algorithm suggests using a TT first, due to its higher sensitivity, and then a confirmatory NTT. This algorithm has higher sensitivity but may yield more false-positive results than the traditional one [11,43,44].
- In the third option, an NTT and a TT are performed at the same time [11].

In the case of a positive TT with a negative NTT, another TT should be performed; if the second treponemal test is negative and the clinical symptoms together with the local epidemiological risk do not suggest syphilis, its diagnosis can be excluded. However, if the result is positive, the patient should either (a) be treated for syphilis if they have no previous history of infection and treatment or (b) repeat the NTT test within the next 2-4 weeks if they have been previously treated for syphilis but have since been re-exposed [11,43,44].

In all cases, a quantitative NTT serum test (VDRL or RPR) should be performed at the beginning of treatment and periodically (one, three and then every six months) after the end of antimicrobial treatment. A four-fold reduction in the serum NTT titer within two years after treatment is desirable, and failure to achieve this reduction is considered therapeutic failure (except for serofast patients, in whom reaching the aforementioned plateau and lack of signs of syphilis should be considered therapeutic success) [6,11,14,43].

Lumbar puncture and the collection of CSF for further testing is recommended only in cases with clinical signs indicative of neurosyphilis, such as cranial nerve dysfunction symptoms, patient’s medical history and reactive treponemal and non-treponemal serological tests. It is not recommended in cases of early syphilis, in asymptomatic neurosyphilis or in cases of non-reactive serological tests (though it may be considered if clinical signs point strongly towards neurosyphilis) [11,43,44].

Possible CSF test results include the following:

- A positive CSF-VDRL result, which in the absence of blood contamination of CSF is diagnostic of neurosyphilis [6,11,43,44].
- A negative CSF-VDRL result, which does not rule out a neurosyphilis diagnosis, due to low sensitivity. When NS is strongly suspected, the test should be followed by a CSF-TT (FTA-ABS is recommended due to 99% sensitivity) [43,50].
- A positive CSF-TT result, which in the case of negative CSF-VDRL confirms a neurosyphilis diagnosis.
- A negative CSF-TT result means the diagnosis of neurosyphilis is unlikely [6,11,43,44].

Lymphocytic pleocytosis (>5 cells/mm^3^) and/or elevated CSF proteins (>45 mg/dL) only support an NS diagnosis due to high sensitivity (95% and 90%, respectively) but low specificity [43,44].

Mononuclear cell count and protein concentration should be tested in the collected CSF specimen, though they may be normal in neurosyphilis. In case they are not, CSF examination must be repeated in 6 weeks to 6 months of treatment [11].

The authors found several inconsistencies in the diagnostic processes presented in several case reports: not all of them were performed in accordance with the mentioned guidelines, and the reasons for them were not disclosed in the reports. It was decided to include them in this article and mention these inconsistencies to emphasize the importance of following the guidelines.

In four cases, the titers of CSF tests associated with the initial NS diagnosis were not provided [33,34], and in two cases, the titers of follow-up tests were not reported; the only available information was that they were reactive, which obviously does not allow for the observation of a four-fold decrease in a quantitative NTT mentioned above [32,34]. Some clinicians opted for RPR tests in CSF in the initial diagnostic process, which resulted in negative results in spite of other tests being positive [38]; that was not the case when CSF-VDRL tests were performed [51]. This confirms that such an approach should be avoided. Additionally, in five cases, both the total CSF protein level and the CSF white blood count were not measured; therefore, it is unknown whether these factors should be additionally monitored after treatment in these patients [31,33,35,36]. As few as three case reports presented diagnostic processes that met the requirements of the guidelines [30,32,37]. It should be noted, however, that certain problems with patient follow-up arose in these three reports: one report did not provide any follow-up [30], another only reported the TT titer (which is likely to remain positive for life and is not recommended for outcome assessment) [6,11,32], and the third one did not specify whether lumbar puncture and CSF collection were performed [37].

Considering the professional literature, the correct use of diagnostic tools appears to be essential for proper monitoring of a patient’s condition after antimicrobial therapy.

### 3.3. Psychiatric Symptoms

Among the psychiatric symptoms presented on admission, the most common were delusions (10/11; 91% of cases) and hallucinations (9/11; 81% of cases), followed by disorientation (5/11; about 42%), sleep disturbances and memory loss (4/11; 36%), attention deficits, aggression, withdrawal and mood disorders such as depression, nervousness and anxiety (3/11; 27%); only one patient reported an instance of illusions. All the patients presented with at least three psychiatric symptoms (four symptoms on average), which stands out from the number of neurological problems reported (Table 3).

### 3.4. Neurological Symptoms

The average number of neurological symptoms was one, with four patients not suffering from any [31,34,36,37] and only one diagnosed with four of them [32]. Interestingly, this patient with neurological disorders was the only one who did not experience improvement after antibiotic therapy. This case and its unsatisfactory resolution are briefly described later in this review. Regarding the symptoms themselves, the most common was dysarthria (three cases), anisocoria with unresponsiveness to light (two cases) and singular reports of bradykinesia, gait disturbance, hearing loss, unilateral positive Babinski sign, neck stiffness and hyperactive tendons (Table 4).

### 3.5. Other Symptoms

There were three cases of fecal and/or urinary incontinence [32,33,37], three cases of weight or appetite loss [32,34,37], and only one patient had a persistent headache [34].

### 3.6. Initial Treatment

Prior to the diagnosis of neurosyphilis, nine out of eleven of the presented patients were treated with the following psychiatric drugs in varying doses, with only modest results or none at all: ziprasidone, aripiprazole, alprazolam, lamotrigine, venlafaxine, quetiapine, diazepam, clozapine, risperidone, olanzapine, lorazepam and divalproex sodium (Table 5).

### 3.7. Treatment of Neurosyphilis—An Antibiotic for Psychosis

The history of syphilis therapy is heavily marked by fire, blood and arsenic. Its origins date back to antiquity, when the ancient Greeks tried to cure diseases with increased temperature (fever), hence the name pyrotherapy—healing with fire or heat [52] This idea was later appreciated and investigated by Julius Wagner-Jauregg, a psychiatrist of Austrian origin, who in his papers reported the varying degrees of success in treating patients with blood infusions from malaria sufferers. Ensuing fever was apparently the cure for psychosis, which had been observed in years prior by the Russian psychiatrist Alexander Rosenblum, who managed to cure 11 out of 22 psychotic patients by inducing fever via several methods in 1876. Some argue that Rosenblum should be regarded as the pioneer of this treatment method, though others solely credit Wagner-Jauregg for the development and application of this ‘malariotherapy’. His first attempts led to the complete recovery of only two of ten neurosyphilitic patients, but later he described treating two hundred patients with a 25% rate of success. The rationale for this controversial method was that malaria could be cured with quinine at the time of experiments, while there was no known cure for syphilis. Wagner-Jauregg was the first psychiatrist to be awarded the Nobel Prize in Physiology or Medicine in 1927 for his discovery of this groundbreaking mode of ‘pyrotherapy’, in other words, healing by fire or heat [53,54,55].

Prior to Jauregg’s malaria-based therapy, the first antimicrobial agent was discovered: Salvarsan (arsphenamine, a trivalent triphenyl and a pentavalent pentaphenyl organoarsenic compound) [56]. It was created and tested in 1910 by Ehrlich, Bertheim and Hata with the intention to treat syphilis and was later found to be effective in treating other diseases. Salvarsan, also known as Compound 606, was proved ineffective in treating tertiary syphilis [57,58,59].

It was not until the introduction of penicillin in 1943 that clinicians treating neurosyphilis (or general paresis, as it was more commonly called) came into possession of a reliable and safe medicine that remains the gold standard in modern times. The current treatment options as follows:

- The first line of NS therapy should be benzyl penicillin in doses as high as 18–24 million units intravenously (IV) a day, divided into 3–4 million units every four hours. This treatment should last 10–14 days [11,60,61].
- Alternatively, 1.2–2.4 million units of procaine penicillin G via intramuscular (IM) injection once a day may be used along with 500 mg of probenecid given orally four times a day. Such a therapeutic approach may be employed if IV penicillin is unavailable or if it is certain that the patient will comply [11,50,61].
- The second-line option is IV injection of 1–2 g of ceftriaxone once daily for 10–14 days, though it is considered neither standardized nor superior to procaine penicillin G [11,61].
- If the patient is allergic to penicillin, they should be desensitized and should undergo the first-line treatment, especially in the case of pregnancy [11,60,61].
- The CDC’s guidelines allow for additional benzathine penicillin treatment after completing the main crystalline or procaine penicillin treatment; in that case, 2.4 million units IM of benzyl penicillin should be administered once a week for up to three weeks to provide an NS treatment of similar length to that of latent syphilis [44].
- Medication other than penicillin and ceftriaxone, such as doxycycline, is currently recommended in patients with bleeding disorders, but only in stages earlier than the tertiary, though according to the WHO 2003 ‘Guidelines for the management of sexually transmitted infections’, doxycycline or tetracycline may be used in treating neurosyphilis in non-pregnant, penicillin-allergic patients. More recent sources disagree [11,60,61].
- Some research suggests that linezolid may be a promising alternative in treatment of NS; it is not yet recommended at any stage of syphilis [62].

Treatment efforts undertaken in the reviewed case reports appear to be more in line with the guidelines than the diagnostic procedures. Out of eleven cases, six were found to comply with literature suggestions, with 1–2 g of ceftriaxone used in three cases, procaine penicillin and probenecid in one, and benzyl penicillin in two cases, of which benzathine penicillin followed in one of them.

Less orthodox treatments included three instances of using benzyl penicillin for more than 10–14 days, prolonging its administration to about three weeks even though benzathine penicillin could have been employed to increase the duration of treatments, which would simplify the process. In one case, 3x1g IM of ceftriaxone once a day was used, and in another one, oral doxycycline 200 mg twice a day was chosen due to the patient’s penicillin allergy (desensitization was not attempted). The treatment regimens of neurosyphilis are presented in Table 6.

### 3.8. Improvements

The extent of improvement in patients’ condition was usually quite satisfactory, considering the infamy of the disease, difficulties in differential diagnosis and the serious initial clinical presentation of the patients. All but one patient experienced at least partial alleviation of symptoms [32]. In the case of four patients, the resolution of symptoms began even before the completion of antimicrobial therapy [30,33,34,36]. Seven patients were reported as free from hallucinations and delusions or experienced them less severely [30,31,33,34,36,38]. Two remained cognitively impaired but otherwise well-functioning [33].

### 3.9. The Outlier: The Case with No Resolution of Symptoms

Only in one case (Toptan et al., 2015 [32]) there was no resolution of any symptoms, and for that reason we would like to describe it in brief. The patient was a man, 40 years of age, with no history of substance abuse. Three years prior to admission he had lost his appetite and willingness to move, two years prior to admission he had become incontinent of urine and feces and developed a speech disorder, and one year prior had become aggressive and began experiencing delusions and hallucinations as well as personality changes. He was finally admitted to a hospital where anisocoria with unresponsiveness to light, dysarthria and bilateral bradykinesia were diagnosed. Serological tests for syphilis were positive, and his CSF was normal except for positive TTs and NTTs: FTA-ABS and VDRL both at 1:2 titers and TPHA at 1:10240 titers. Syphilis was diagnosed, and benzyl penicillin at a dose of 24 million units was administered via IV injection for 21 days, but the patient experienced no resolution of symptoms. An additional diagnosis of organic brain syndrome was later made due to the lack of improvement after antimicrobial therapy; global cerebral and cerebellar atrophy and signs of past trauma of unknown origin in the right parietal lobe were found. This case stands out from others in that the patient presented four neurological symptoms, all of them suggesting a seriously damaged CNS, without any improvement after syphilis treatment and additional psychiatric treatment (risperidone 3 mg/day, haloperidol 10 mg/day and carbamazepine 800 mg/day).

### 3.10. Neurosyphilis Battle Efforts

Seeing the severity of neurosyphilis, it is of utmost importance to take action against this disease. The authors of this study believe that their home country, Poland, has implemented a holistic model that is still being improved upon. In this model, the diagnostic procedure of syphilis may begin in the office of a family medicine doctor, who is usually the primary care provider and is free to refer the patient for a serum VDRL test free of charge [63]. Once a definitive diagnosis of syphilis is made, treatment becomes mandatory [64]. Alternatively, a patient can visit one of the Consulting-Diagnostic Points to undergo syphilis (but also HIV and HCV) testing free of charge and anonymously [65]. Pregnant women are provided with repeated prenatal tests, which include VDRL [66]. However, what still needs to be improved upon is raising awareness of sexually transmitted diseases and their possible complications. This should be implemented at many levels, including school education and social campaigns.

## 4. Conclusions

The main goal of this systematic review was to determine the profile of the average patient with neurosyphilis and, based on the authors’ findings, the following can be concluded regarding the average patient:

- Most likely suffers from delusions, hallucinations, is disoriented and has difficulty sleeping;
- May present dysarthria or anisocoria;
- May have a normal level of protein and white blood cells in CSF;
- Will not benefit from standard psychiatric treatment administered before antimicrobial treatment;
- Will likely experience symptom alleviation from antimicrobial therapy, even if the therapy itself is not precisely following guidelines;
- May be left with cognitive impairment after the therapy is concluded.

Another reason for concern in the case of neurosyphilis is the age of the patient. In elderly patients, many symptoms may be incorrectly attributed to such diseases as Alzheimer’s or Parkinson’s disease, typically expected in this age group. It may be the case that such patients will not be willing to share details of their sexual history, due to shame or stigma. Conversely, neurological manifestations of syphilis, which can take up to decades, in a person seemingly too young to display signs of a venereal disease may be indicative of sexual abuse. Evidence of this was found in two rejected case reports [67,68]. The experience of such abuse, if shared during the interview with a patient showing appropriate symptoms, may lead the clinician to suspect neurosyphilis.

Due to the insufficient number of case reports on psychoses secondary to neurosyphilis available in databases, it is unlikely that any statistical analysis will provide significant relationships between patients’ parameters and their treatment outcomes; therefore, further research is required. Perhaps a review covering countries beyond Europe will solve the issue, although concerns about local variability of the disease may be raised.

Another issue regarding the reviewed case reports is the quality of data, as seen above in the JBI Checklist; several authors did not fully disclose the antimicrobial therapy, leaving the reader to speculate on the dosage and duration of treatment, both of which may be useful in the context of data collection and analysis. Additionally, more information on the passage of time in relation to patients’ treatment would allow for a better understanding of the dynamics of diagnostic and medical processes. Knowing how long a patient stays at the ward and how long treatment was given before proper diagnosis could provide another clue towards a potential suspicion of neurosyphilis, along with antipsychotic resistance, mean age and symptoms.

More standardized diagnostic approaches should be applied. Correctly reported and archived titers, along with the selection of methods more consistent with guidelines, appear to be essential for appropriate follow-up testing and for more convincing diagnoses. Implementing such a model and sharing this information in future reports may provide sufficient data to analyze potential associations between patients’ test results and their prognosis.

A disease as complex and partly forgotten as neurosyphilis requires great awareness and cooperation between various medical specialties. Despite its mimicry and variability in presentation, the discussed case reports prove that it can be successfully dealt with.

## Figures and Tables

**Table 1:** Results of systematic search of case reports; articles accepted for further appraisal.

№	Authors and Reference	Report Title
1.	De Bruijn et al., 2017 [27]	‘The big imitator strikes again a case report of neurosyphilis in a patient with newly diagnosed HIV’
2.	Brooke et al., 1987 [28]	‘Neurosyphilis—a treatable psychosis’
3.	Blažeković et al., 2018 [29]	‘Neurosyphilis: The shape of a rising threat’
4.	Turan et al., 2007 [30]	‘Effectiveness of olanzapine in neurosyphilis related organic psychosis: a case report’
5.	Noblett, Roberts, 2015 [31]	‘The importance of not jumping to conclusions: syphilis as an organic cause of neurological, psychiatric and endocrine presentations’
6.	Toptan, 2015 [32]	‘Neurosyphilis: a case report’
7.	Akinci et al., 2016 [33] *	‘Neurosyphilis in psychiatric settings: three case reports’
8.	Kararizou et al., 2006 [34]	‘Psychosis or simply a new manifestation of neurosyphilis?’
9.	Friedrich et al., 2009 [35]	‘Manifest psychosis in neurosyphilis’
10.	Güler, Leyhe, 2011 [36]	‘A late form of neurosyphilis manifesting with psychotic symptoms in old age and good response to ceftriaxone therapy’
11.	Murtza et al., 2022 [37]	‘A case of neurosyphilis with psychosis and hippocampal atrophy’
12.	Toffanin et al., 2019 [38]	‘A case report of neurosyphilis limbic encephalitis with reversible Geschwind syndrome and mood disorder’

Twelve case reports met the inclusion criteria. The article marked with * presented more than one case.

**Table 2:** JBI Checklist for Case Reports.

№	Case Report Authors and Reference (Case Number)	Q1	Q2	Q3	Q4	Q5	Q6	Q7	Q8	Overall Appraisal
1.	De Bruijn et al., 2017 [27]	^+^	^+^	^+^	^+^	^−^	^−^	^+^	^+^	rejected
2.	Brooke et al., 1987 [28]	^+^	^+^	^+^	^−^	^−^	^+^	^+^	^+^	rejected
3.	Blažeković et al., 2018 [29]	^+^	^+^	^+^	^+^	^−^	^−^	^−^	^+^	rejected
4.	Turan et al., 2007 [30]	^+^	^+^	^+^	^+^	^+^	^−^	^+^	^+^	approved
5.	Noblett, Roberts, 2015 [31]	^+^	^+^	^+^	^+^	^+^	^−^	^+^	^+^	approved
6.	Toptan, 2015 [32]	^+^	^+^	^+^	^+^	^+^	^+^	^+^	^+^	approved
7.	Akinci et al., 2016 (Case 1) [33]	^+^	^+^	^+^	^+^	^+^	^+^	^−^	^+^	approved
8.	Akinci et al., 2016 (Case 2) [33]	^+^	^+^	^+^	^−^	^+^	^+^	^−^	^+^	approved
9.	Akinci et al., 2016 (Case 3) [33]	^+^	^+^	^+^	^+^	^+^	^+^	^−^	^+^	approved
10.	Kararizou et al., 2006 [34]	^+^	^+^	^+^	^+^	^+^	^+^	^+/−^	^+^	approved
11.	Friedrich et al., 2009 [35]	^+^	^+^	^+^	^+^	^+^	^+^	^+/−^	^+^	approved
12.	Güler, Leyhe, 2011 [36]	^+^	^+^	^+^	^+^	^+^	^+^	^+^	^+^	approved
13.	Murtza et al., 2022 [37]	^+^	^+^	^+^	^+^	^+^	^+^	^+/−^	^+^	approved
14.	Toffanin et al., 2019 [38]	^+^	^+^	^+^	^+^	^+^	^+^	^+/−^	^+^	approved

Q1: Were patient’s demographic characteristics clearly described? Q2: Was the patient’s history clearly described and presented as a timeline? Q3: Was the current clinical condition of the patient on presentation clearly described? Q4: Were diagnostic tests or assessment methods and the results clearly described? Q5: Was the intervention(s) or treatment procedure(s) clearly described? Q6: Was the post-intervention clinical condition clearly described? Q7: Were adverse events (harms) or unanticipated events identified and described? Q8: Does the case report provide takeaway lessons? (+) yes, (+/−) unclear, (−) no.

**Table 3:** Psychiatric symptoms observed in patients, their number per patient and frequency.

Observed Symptoms	Turan et al., 2007 [30]	Noblett, Roberts, 2015 [31]	Toptan, 2015 [32]	Akinci et al., 2016 (Case 1) [33]	Akinci et al., 2016 (Case 2) [33]	Akinci et al., 2016 (Case 3) [33]	Kararizon et al., 2006, [34]	Friedrich et al., 2009 [35]	Guller, Leyhe, 2011 [36]	Murtza et al., 2022 [37]	Toffanin et al., 2019 [38]	Fraction of Patients with the Symptoms
Anxiety/depression/nervousness	1							1	1			0.27
Hallucinations	1	1	1	1		1	1		1	1	1	0.82
Illusions			1									0.09
Sleep disturbance	1			1				1			1	0.36
Delusions	1	1		1	1	1	1	1	1	1	1	0.91
Attention deficits				1			1	1				0.27
Aggression		1	1			1						0.27
Memory loss (short or long term)			1		1			1		1		0.36
Disorientation				1	1	1	1	1				0.45
Withdrawal						1	1			1		0.27
Number of symptoms	4	3	4	5	3	5	5	6	3	4	3	

Blank space—symptom not present, ‘1’—symptom present.

**Table 4:** Neurological symptoms observed in patients, their frequency and quantity per patient.

Observed Symptoms	Turan et al., 2007 [30]	Noblett, Roberts, 2015 [31]	Toptan, 2015 [32]	Akinci et al., 2016 (Case 1) [33]	Akinci et al., 2016 (Case 2) [33]	Akinci et al., 2016 (Case 3) [33]	Kararizou et al., 2006 [34]	Friedrich et al., 2009 [35]	Güler, Leyhe, 2011 [36]	Murtza et al., 2022 [37]	Toffanin et al., 2019 [38]	Number of Patients Presenting with a Symptom
Hearing loss	1											1
Dysarthria	1		1		1							3
Bradykinesia and/or bradylalia			1									1
Anisocoria			1								1	2
No response to light			1								1	2
Unstable gait						1						1
Neck stiffness				1								1
Hyperactive tendons						1						1
Babinski sign								1				1
Number of symptoms in a patient	2	0	4	1	1	2	0	1	0	0	2	

Blank space—symptom not present, ‘1’—symptom present.

**Table 5:** Initial treatment of patients (prior to syphilis diagnosis).

References	Treatment Prior to Neurosyphilis Diagnosis
Turan et al., 2007 [30]	Ziprasidone, 20 mg, twice a day Olanzapine velotab, 10 mg, twice a day
Noblett, Roberts, 2015 [31]	Aripiprazol, 15 mg, once a day Aripiprazol, 400 mg, once a month, intramuscularly
Toptan, 2015 [32]	None
Akinci et al., 2016 (Case 1) [33]	Aripiprazol, 15 mg, once a day + alprazolam, 0.5 mg once a day + lamotrigine, 100 mg, once a day Venlafaxine, 150 mg, once a day + quetiapine, 50 mg, once a day
Akinci et al., 2016 (Case 2) [33]	Diazepam, 40 mg, once a day + quetiapine, 300 mg, once a day
Akinci et al., 2016 (Case 3) [33]	None
Kararizou et al., 2006 [34]	Risperidone, 10 mg, once a day Haloperidol, 30 mg, once a day Clozapine, 500 mg, once a day
Friedrich et al., 2009 [35]	Olanzapine, 25 mg + lorazepam, 8 mg * Olanzapine, 25 mg + lorazepam, 8 mg + quetiapine, 500 mg + trazodone, 150 mg *
Güler, Leyhe, 2011 [36]	Risperidone, 1 mg, once a day
Murtza et al., 2022 [37]	Risperidone *
Toffanin et al., 2019 [38]	Olanzapine, 20 mg, once a day + diazepam, 8 mg, once a day Olanzapine, 10 mg, once a day + divalproex sodium, 1000 mg, once a day + diazepam, 6 mg, once a day

Each therapeutic attempt is denoted by a dot. Drugs administered in conjunction (polytherapy) are denoted by a plus. * No more data was available in the report.

**Table 6:** Administered antimicrobial treatment of neurosyphilis.

References	Drug	Dosage	Duration
Turan et al., 2007 [30]	Ceftriaxone IM	1 g per day	15 days
Noblett, Roberts, 2015 [31]	Doxycycline p.o.	200 mg twice a day	28 days
Toptan, 2015 [32]	Benzylpenicillin	24 million units per day	21 days
Akinci et al., 2016 (Case 1) [33]	Ceftriaxone IM	1 g thrice a day	14 days
Akinci et al., 2016 (Case 2) [33]	Benzylpenicillin	24 million units per day	21 days
Akinci et al., 2016 (Case 3) [33]	Benzylpenicillin	24 million units per day	20 days
Kararizou et al., 2006 [34]	Benzylpenicillin	24 million units per day	14 days
Friedrich et al., 2009 [35]	Benzylpenicillin; benzathine benzylpenicillin	24 million units per day; 2.4 million units per week	14 days; 3 weeks
Güler, Leyhe, 2011 [36]	Ceftriaxone	2 g per day	14 days
Murtza et al., 2022 [37]	Procaine penicillin + probenecid	12.4 million units per day + 500 mg four times a day	14 days
Toffanin et al., 2019 [38]	Ceftriaxone	1 g per day	14 days

(+) denotes medicine used in conjunction, ‘;‘ denotes medicine used in sequence, IM—intramuscularly, p.o.—per os.

## Data Availability

No new data were created or analyzed in this study. Data sharing is not applicable to this article.

## Supplementary Materials

The following supporting information can be downloaded at: https://www.mdpi.com/article/10.3390/pathogens13110959/s1, Table S1: Preferred Reporting Items for Systematic reviews and Meta-Analyses extension for Scoping Reviews (PRISMA-ScR) Checklist [69].
